# 

**Published:** 1873-08

**Authors:** 


### TABLE OF CONTENTS.

### ORIGINAL COMMUNICATIONS.

THE ORIGIN, NATURE AND TREATMENT OF FEVER, ETC. By CH. RAUSCHENBERG, M.D........243
TWO CASES OF CYSTS OF THE IRIS, ETC. By A. W. CALHOUN, M.D........................255
SACCHARATED CALOMEL. By ROBERT BATTEY, M.D........................................261
THE THERAPEUTIC EFFECTS AND USES OF MERCURY, ETC. By WM. H. DOUGHTY, M.D........265
REPORT OF AMPUTATION OF THE THIGH, ETC. By DE SAUSSURE FORD, M.D..................276

### SELECTIONS.

OBSERVATIONS UPON TREATMENT OF YELLOW FEVER. By JOSEPH JONES, M.D...............280

CHOLERA.........................................................................287

GONORRHŒA OF THE RECTUM.........................................................289

### EDITORIAL AND MISCELLANEOUS.

NEW ORLEANS—CLIMATIC, HYGIENIC, MEDICAL..........................................290
A NIGHT IN THE MOUNTAINS.........................................................294
THE MEDICAL EXPERTS IN THE VAN NESS-WHARTON TRIAL................................298
NOSTRUM QUACKERY.................................................................300
CHOLERAIC DIARRHŒA...............................................................301

